# Haplotype Diversity and Reconstruction of Ancestral Haplotype Associated with the c.35delG Mutation in the GJB2 (Cx26) Gene among the Volgo-Ural Populations of Russia

**Published:** 2011

**Authors:** L.U. Dzhemileva, O.L. Posukh, N.A. Barashkov, S.A. Fedorova, F.M. Teryutin, V.L. Akhmetova, I.M. Khidiyatova, R.I. Khusainova, S.L. Lobov, E.K. Khusnutdinova

**Affiliations:** Institute of Biochemistry and Genetics, Ufa Research Center, Russian Academy of Sciences; Institute of Cytology and Genetics, Siberian Branch, Russian Academy of Sciences; Yakut Research Center of Complex Medical Problems, Siberian Branch, Russian Academy of Medical Sciences

**Keywords:** hereditary nonsyndromic sensorineural hearing loss, *GJB2*(Cx26) gene, c.35delG mutation, ancestral haplotype, populations of the Volga-Ural region

## Abstract

The mutations in the*GJB2*(Сх26) gene make the biggest contribution to hereditary hearing loss. The spectrum and prevalence of the*GJB2*gene mutations are specific to populations of different ethnic origins. For several*GJB2 *mutations, their origin from appropriate ancestral founder chromosome was shown, approximate estimations of “age” obtained, and presumable regions of their origin outlined. This work presents the results of the carrier frequencies’ analysis of the major (for European countries) mutation c.35delG (*GJB2*gene) among 2,308 healthy individuals from 18 Eurasian populations of different ethnic origins: Bashkirs, Tatars, Chuvashs, Udmurts, Komi-Permyaks, Mordvins, and Russians (the Volga-Ural region of Russia); Byelorussians, Ukrainians (Eastern Europe); Abkhazians, Avars, Cherkessians, and Ingushes (Caucasus); Kazakhs, Uzbeks, Uighurs (Central Asia); and Yakuts, and Altaians (Siberia). The prevalence of the c.35delG mutation in the studied ethnic groups may act as additional evidence for a prospective role of the founder effect in the origin and distribution of this mutation in various populations worldwide. The haplotype analysis of chromosomes with the c.35delG mutation in patients with nonsyndromic sensorineural hearing loss (N=112) and in population samples (N =358) permitted the reconstruction of an ancestral haplotype with this mutation, established the common origin of the majority of the studied mutant chromosomes, and provided the estimated time of the c.35delG mutation carriers expansion (11,800 years) on the territory of the Volga-Ural region.

## INTRODUCTION

Hereditary deafness is a frequent disorder in humans: it is recorded in 1/1,000 newborns. The etiology and pathogenesis of this disease are still to be clarified; however, approximately half of the cases of hereditary deafness are a result of genetic disorders [1].

The hereditary forms of innate hearing loss are characterized by clinical polymorphism and genetic heterogeneity. In the nuclear genome, about 114 loci were mapped and 55 genes were identified, whose mutations to a certain extent cause hearing loss. About 80% of all cases of hereditary nonsyndromic hearing loss fall within the category of autosomal-recessive forms; 15–20% – autosomal-dominant, and about 1% - the form linked with the Х-chromosome and mitochondrial forms of deafness [2].

**Fig. 1:**
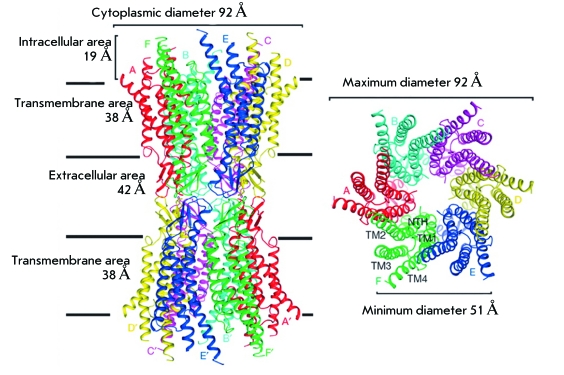
Structure of intercellular channels formed by molecules of connexin 26. A, B, C, D, F, E and A’, B’, C’, D’, F ‘, E’ - connexin 26 molecules in connexones of neighboring cells; TM 1-4 – transmembrane protein segments of Cx26; NTH - N-terminal helix of protein Cx26. Figure was adapted from [3] by permission from Macmillan Publishers Ltd.

The most frequent cause of nonsyndromic autosomal recessive hearing loss in humans is the mutations in the *GJB2* gene (gap junction β2, subunit β2 of the gap junction protein), localized in the chromosomal region 13q11–q13 and coding connexin 26 (Сх26), which is the transmembrane protein involved in the formation of connexons. Connexons are structures consisting of six protein subunits, which form cellular channels, ensuring a fullblown ion exchange among adjacent cells. This facilitates the maintenance of the homeostasis of endolymph in cochlea tissues. Recently, the fine structure of intercellular channels formed by connexin 26 was reported ( *Fig. 1* ) [3]. When there are defects in connexin 26, the functioning of the intercellular channels is irreversibly disrupted in the tissues of the internal ear and endolymph homeostasis is not restored: a factor that is necessary for normal sound perception [4].

So far, in the *GJB2* gene *, * over 150 pathogenic mutations (mainly recessive), several polymorphisms and sequencing variants whose role in the pathogenesis of hearing loss is still unclear, have been described [2]. The spectrum and frequencies of the *GJB2 * gene mutations are characterized by significant interpopulation differences. The racial and/or ethnic specificity of the spread of several *GJB2* gene mutations is preconditioned in some cases by the founder effect, as well as by, in all likelihood, the geographic and social isolation of some populations. Researchers have managed to show the origin of some *GJB2 * gene mutations from an ancestor founder chromosome, to obtain approximate estimations of the “age” of the mutations, and to outline suggested regions where they appeared [5–10]. The mutation c.35delG (p.Gly12Valfsx1) is the most frequent in Europe. It first emerged, according to various estimates, 10,000 to 14,000 years ago on the territory of the Middle East or Mediterranean region (probably on the territory of modern Greece) [10–12] and spread in Europe with the migrations of the neolithic population of *Homo sapiens* [6]. Haplotype analysis of the chromosomes with mutation c.235delC (p.Leu79Cysfsx3) in populations of Japan, Korea, China, and Mongolia has allowed to put forward a hypothesis about the founder effect regarding the origin and distribution of this mutation in East Asia and to estimate its “age” (~11,500 years) and presumed appearance in the Lake Baikal region, from where it spread, by way of sequential migrations, over Asia [7]. The “age” of the mutation c.71G>A (p.Trp24X), most widespread in India, has also been assessed (~7,880 years) [8]. The ethnic specificity of the mutation c.167delT (p.Leu56Argfsx26) and mutation c.427C > T (p.Arg143Trp) was shown for populations of the Ashkenazi Jews [5] and for some populations of Western Africa (Ghana) [13, 14], respectively. 

In Russia, several research groups have studied the hereditary forms of deafness [15–32]. In most cases, they have considered the genetic-epidemiological and clinical-genetic peculiarities of the inherited forms of hearing loss, and a series of papers are devoted to the molecular-genetic analysis of the *GJB2 * gene or its single mutations [17–21, 25–30]. Some authors have obtained data on the specificity of the range and frequency of separate mutations in the *GJB2 * gene function in the studied region. For instance, the most frequent mutations in the Siberian populations (Yakuts and Altaians) are IVS1 + 1G>A [27] and c.235delC [25], respectively. The populations of the Volga-Ural region, as generally in the European part of the continent, predominantly exhibit the mutation c.35delG [18–22, 28]. Local differences in the carrier frequency of the mutation c.35delG are probably related to the genetic history of some populations, and factors of population dynamics, and migration routеs of the с.35delG carriers in the world. The available data on the contribution of the *GJB2 * mutations to the development of a pathology in patients with nonsyndromic sensorineural hearing loss (NSHL), living in the Volga-Ural regions, and the population-based data on the carrier frequency of the most significant recessive mutation c.35delG permitted an adequate assessment of the haplotypic diversity of the chromosomes bearing the mutation с.35delG, the reconstruction of the possible ancestor haplotype linked to that mutations, and the estimated time of its appearance on the territory of the Volga-Ural regions, which represents the eastern border of the habitat of the mutation с.35delG.

## EXPERIMENT

**Fig. 2:**
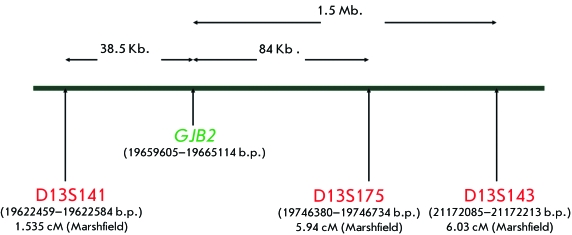
Localization of the microsatellite markers D13S141, D13S175, and D13S143, flanking the *GJB2* gene, on chromosome 13. The distance between the *GJB2* gene and the markers is indicated by arrows.

The material taken for the haplotypic analysis and estimates of the “age” of c.35delG of the gene *GJB2 * was 56 DNA samples (112 chromosomes) obtained from patients with NSHL, residing in the Volga-Ural regions, in which the mutation.35delG was identified in the homozygote state (32 Russians, 10 Tatars, 1 Bashkirs, 4 Ukrainians, 2 Armenians, and 7 individuals of mixed ethnicity). The control group included 179 (358 chromosomes) healthy individuals from three ethno-geographical groups of the Russians ( *N* = 86); Tatars ( *N* = 62); and Bashkirs ( *N* = 31) without this mutation.

To analyze the carrier frequency of c.35delG 2,308 DNA samples were used, which were obtained from healthy individuals-representatives of different populations of the Volga-Ural region, Central Asia, Northern Caucasus, Eastern Europe, and Siberia, belonging to four language families ( *Table 1* ). 

The blood samples were obtained during expeditions between the years of 2000 and 2010, after receiving the informed written consent of the participants in the study. The genomic DNA was extracted from peripheral blood using the phenol-chloroform extraction method.

The present scientific-research work was approved by the local committee for biomedical ethics at the IBG UNT of the Russian Academy of Sciences (Ufa).

**Screening of the с.35delG mutation in the**

*GJB2* **gene**

The screening of the mutation с.35delG in the *GJB2* genewas carried out with the allele-specific amplification of fragments of the coding region of the *GJB2 * geneusing the primers listed in *Table 2* . The results were visualized by vertical electrophoresis in a 10% polyacrylamide gel (PAAG) with further staining of the ethidium bromide solution in the standard concentration and viewing in ultraviolet rays.

**Analysis of haplotypes and estimate of the “age” of the mutation c.35delG**

For the analysis of the haplotypes and the estimated age of the mutation c.35delG in the *GJB2* gene *,* three high-polymorphic microsatellite СА-markers were used: D13S175, D13S141, and D13S143 [6, 9, 10, 12, 36], flanking the DFNB1 locus, which contains the *GJB2 * gene. The physical and genetic localization of the markers at chromosome 13 and genetic distances between them, as well as the *GJB2* gene *, * were identified on the basis of the Marshfield genetic linkage map (http://www.ncbi.nlm.nih.gov/mapview/). The total physical distance of the flanked region was ~ 2  Mb. ( *Fig. 2* ). The choice of the markers was preconditioned to fall with the strive to get the possibility of comparable data, as earlier these markers were used for the assessment of the age of mutations in the gene *GJB2 * in different populations [6, 9, 10, 12, 36]. 

**Table 1:** Carrier frequencies of the c.35delG mutation in 18 ethnic groups dwelling on the territory of Eurasia

Population	Linguistic affiliation (group)	Region	N	Number of heterozygote carriers of the mutation c.35delG / total numbers of individuals tested(carrier frequency)
Eastern Europe
Byelorussians	Indo-European / Slavic	The Republic of Belarus (disperse sample)	97	6/97(0.062)
Ukrainians	Indo-European / Slavic	Kharkov region and Poltava region, Ukraine	90	3/90(0.033)
Volga-Ural region
Russians	Indo-European / Slavic	Yekaterinburg, the Russian Federation (RF)	92	2/92(0.022)
Bashkirs	Altaic / Turkic	Baimakskii, Burzyanskii, Abzelilovskii, Kugarchinskii, Salavatskii, and Arkghangelskii districts, the Republic of Bashkortostan, RF	400	1/400(0.003)
Tatars	Altaic / Turkic	Al’met’evskii and Yelabuzhskii districts, the Republic of Tatarstan, RF	96	1/96(0.010)
Chuvashs	Altaic / Turkic	Morgaushskii district, the Chuvash Republic, RF	100	0/100
Mordvins	Uralic / Finno-Ugric	Staroshaiginskii district, the Republic of Mordovia, RF	80	5/80(0.062)
Udmurts	Uralic / Finno-Ugric	Malopurginskii district of the Udmurt Republic and Tatyshlinskii district of the Republic of Bashkortostan, RF	80	3/80(0.037)
Komi-Permyaks	Uralic / Finno-Ugric	Kachaevskii district, the Komy-Permyak Autonomous District, RF	80	0/80
Central Asia
Kazakhs	Altaic / Turkic	Alma-Atinskaya region, Kyzylordinskaya region, and Abaiskii district, Kazakhstan	240	2/240(0.008)
Uighurs	Altaic / Turkic	Alma-Atinskaya region, Kazakhstan	116	1/116(0.009)
Uzbeks	Altaic / Turkic	The Republic of Uzbekistan (disperse sample)	60	0/60
Caucasus
Abkhazians	North-Caucasian / Adygo-Abkhaz	Abkhazia and Georgia (disperse sample)	80	3/80(0.038)
Avars	North-Caucasian / Dagestan	Gumbetovskii district, the Republic of Dagestan, RF	60	0/60
Cherkessians	North-Caucasian / Adygo-Abkhaz	The Karachaevo-Cherkess Republic, RF	80	1/80(0.013)
Ingushes	North-Caucasian / Nakh	Nazran’ district, the Republic of Ingushetia, RF	80	0/80
Siberia
Altaians	Altaic / Turkic	The Republic of Altai, RF	230	0/230
Yakuts	Altaic / Turkic	Megino-Kangalasskii, Amginskii, Churapchinskii, Tattinskii, Verkhnevilyuiskii, Vilyuiskii, Nyurbinskii, and Suntarskii uluses (districts), the Republic of Sakha (Yakutia), RF	247	1/247(0.004)

The STR-markers were genotyped using PCR at the thermocycler (Eppendorf), and appropriate oligonucleotide primers were used ( *Table 2* ). Products of the PCR-reaction were separated by vertical electrophoresis (glass size 20 × 20 cm, Helicon, Russia) in 10% PAAG and 5% glycerin. The gels were stained with silver ions. 

**Table 2:** Sequences of primers used for amplification

Locus	Name and nucleotide sequence of primers	Detection method	Reference
*GJB2*(13q11-q12)	35delG F 5’-CTTTTCCAGAGCAAACCGCCC-3’35delG R 5’-TGCTGGTGGAGTGTTTGTTCAC-3’	Visualization of PCR fragments in 10% PAAG	[15]
D13S141	F- 5’-GTCCTCCCGGCCTAGTCTTA-3’R-5’-ACCACGGAGCAAAGAACAGA-3’		[33]
D13S143	F-5^’^-CTC ATG GGC AGT AAC AAC AAAA-3^’^R-5^’^-CTT ATT TCT CTA GGG GCC AGC T-3^’^		[34]
D13S175	F-5^’^-TAT TGG ATA CTT GAA TCT GCT G-3^’^R-5^’^-TGC ATC ACC TCA CAT AGG TTA-3^’^		[35]

The linkage disequilibrium between alleles of the 13th-chromosome loci was calculated using the following formula: 

δ = ( *Pd* – *Pn* )/(1 – *Pn* ),

where δ is the measure of linkage disequilibrium, *Pd * is the frequency of the associated allele among mutant chromosomes, and *Pn* is the frequency of the same allele among intact chromosomes [37].

The statistical significance of differences in the frequencies of the alleles of the studied markers on 112 chromosomes containing c.35delG and 358 chromosomes without this mutation was assessed using the standard χ ^2 ^ test 2х2 (the MedStat software).

The age of expansion of the founder haplotype bearing the mutation с.35delG in the *GJB2 * gene was estimated via the genetic clock approach [38], which is based on the definition of the number of generations ( *q* ) from the moment of the mutation appearance in the population to the present, proceeding from the ratio of linkage disequilibrium in terms of polymorphous markers linkage with the locus of the disorder. This age was calculated using the following formula:

*q* = log[1 – *Q* /(1 – *Pn* )]/log(1 – Ө),

where *q * is the number of generations since the moment the mutation appeared in the population, *Q * is the share of mutant chromosomes without the founder haplotype, *Pn * is the frequency of the allele of the founder haplotype in the population, and Ө is the recombinant fraction. The value of Ө was computed given the physical distance of the markers from the location of the mutation, stemming from the ratio 1 cM = 1,000,000 b.p.

The value for the allele association was estimated by the coefficient of standard linkage disequilibrium according to [39]:

**Figure:**
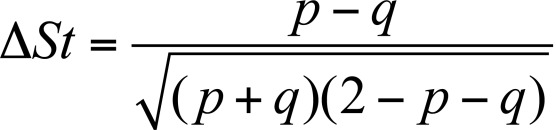


where *р * and *q * are the frequencies of the alleles or haplotypes of the normal ( *р* ) and mutant ( *q* ) chromosomes.

The haplotypic diversity indicator equivalent to the expected heterozygosity was calculated using the formula:

**Figure:**
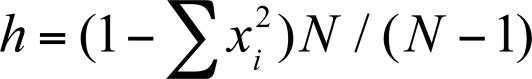


where *х * is the frequency of each haplotype in the population, and *N * is the sample size [40].

## RESULTS AND DISCUSSION

In many populations worldwide, the main contributor to the development of nonsyndromic sensorineural hearing loss (NSHL) is the mutations of the *GJB2* gene. In most European populations, up to 40–50% of cases of NSHL are preconditionally caused by one of the major recessive mutations of this gene, c.35delG, which is revealed in the homozygous or compound-heterozygous state [41]. In connection with this, many researchers have analyzed the carrier frequency of c.35delG in a variety of populations in the world. A large-scale study which embraced 17 European countries showed that the mean carrier frequency of the c.35delG mutation amounts to 1.96% (1/51), ranging from 2.86% (1/35) in South-European countries to 1.26% (1/79) in Northern Europe [42]. In the Mediterranean region, the highest carrier frequency of c.35delG were observed in Greece (3.5%), in the southern regions of Italy (4.0%), and in France (3.4%) [43]. As a result of the meta-analysis of the c.35delG carrier frequency in over 23,000 individuals from different populations, carried out on the basis of the data published between the years of 1998 and 2008, the average regional frequencies of c.35delG were determined in the European (1.89%), American (1.52%), Asian (0.64%), and African (0.64%) populations, as well as in Oceania (1%). Also, the decreasing gradient of the carrier frequency of c.35delG (from 2.48 to 1.53%) from south to north in European populations and from west to east (from 1.48 to 0.1%) in Asian populations [44] was confirmed.

**Carrier frequency of the mutation c.35delG**

**Fig. 3:**
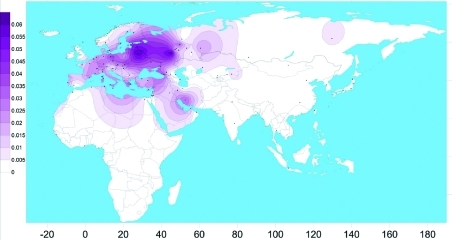
The spatial distribution of the carrier frequency of the c.35delG mutation in the *GJB2* gene in the populations of Eurasia (performed using the program SURFER 9.0 Golden Software Ink).

An analysis of the carrier frequency of the mutation c.35delG in 18 populations of Russia and the former Soviet states was carried out ( *Table 1* ). 

High carrier frequencies of the c.35delG were revealed in two Eastern European populations: Ukrainians (3.3%) and Byelorussians (6.2%). In the Turkic-speaking populations of the Volga-Ural region, the carrier frequencies of the mutation с.35delG were 1.0, 0.3, and 0% in Tatars, Bashkirs and Chuvashs, respectively. In the Finno-Ugric populations of the Volga-Ural region, the с.35delG mutation was present with a high carrier frequency of 6.2% in Mordvins, 3.7% in Udmurts, and it was absent in Komi-Permyaks. These frequency fluctuations among the studied populations of the Volga-Ural region are likely due to the specific features of the historic development of these populations in the region, or could be the consequence of the relatively small size of the samples. Earlier, a high carrier frequency of с.35delG (4.4%) was found in Estonians, an apparent exception for Northern European populations, who typically have low frequencies of с.35delG [42]. These data, as well as the data obtained during other studies [15, 20–22, 28, 29], indicate the significant variation in the carrier frequency of с.35delG among indigenous populations of the Volga-Ural region. The carrier frequency of the с.35delG, singled out in Russians (2.2%), was comparable to the results for the Russian population in the Central region of Russia [15, 18, 28]. In the Turkic-speaking populations of Central Asia (Kazakhs, Uighurs, and Uzbeks) the mutation c.35delG with a low carrier frequency was observed in Kazakhs (0.8%), Uighurs (0.9%), and it was absent in Uzbeks. In the Turkic-speaking populations of Siberia (Yakuts and Altaians) a relatively low carrier frequency of the mutation c.35delG (0.4%) was revealed in the Yakut population but was not detected in Altaians. The North Caucasus region was in the past one of the crucial migration corridors in Eurasia. It is characterized by a high diversity of the population and a complex historical development of its resident ethnoses. In the North Caucasus populations (Abkhazians, Avars, Cherkessians, and Ingushes), the mutation с.35delG was discovered only in Abkhazians (3.8%) and Cherkessians (1.3%).

The spatial distribution of the carrier frequency of the mutation in the Eurasian populations obtained on the basis of our own data and that of relevant published information on the c.35delG available as of 2010 [24] is presented in *Fig. 3.*

The data obtained significantly added to the picture of the mutation c.35delG distribution in Eurasia: European regions of Russia, just as the European part of the continent on the whole, are characterized by a high frequency of с.35delG, and this mutation is widespread in the polyethnic population of the Volga-Ural region. However, the mechanisms of its spreading and time of appearance in the Volga-Ural region are yet to be studied. To answer these questions, we carried out a haplotypic analysis of the chromosomes carrying c.35delG and those without it, using three high-polymorphous microsatellite СА-markers: D13S175, D13S141, and D13S143 [6, 9, 10, 12, 36], which flank the locus DFNB1 containing the *GJB2* gene ( *Fig. 2* ). 

**Frequencies of alleles of loci D13S141, D13S175, and D13S143**

*Table 3*lists the distribution of frequencies for the alleles of microsatellite markers D13S141, D13S175, and D13S143 in the NSHL patients (c.35delG-mutant chromosomes) and in the control sample (normal chromosomes), including three ethnic groups (Russians, Tatars, and Bashkirs).

In other studies, the area sized 2  Mb and covered by these three markers allowed for the calculation of the approximate number of past generations since the start of the expansion of the proposed founder haplotype, including mutations of the *GJB2* gene, in the populations of India (mutation p.Trp24X) [8] and Morocco (c.35delG) [9]. A panel consisting of 8 STR-markers (D4S189, D13S1316, D13S141, D13S175, D13S1853, D13S143, D13S1275, and D13S292) and 2 SNP-markers was applied in dating the splicing site IVS1 + 1G>A mutation of the *GJB2* genein the Yakut population [27]. The age of the mutations c.35delG and с.235delC was computed using the 6 SNP-markers [6, 7]. In later studies aimed at clarifying the age of the mutation c.35delG in Greece, two STR-markers, D13S175 and D13S141, and six SNP-markers were used [12].

**Table 3:** Distribution of allele frequencies of microsatellite markers D13S141, D13S175, and D13S143 in patients with NSHL (chromosomes with the c.35delG mutation) and in the control sample (normal chromosomes)

Allele (b.p.)	Chromosomes with c.35delG mutation (N=112)		Normal chromosomes(N=358)		χ^2^	Р(95% significance level)
Number of chromosomes	Allele frequency	Number of chromosomes	Allele frequency
D13S141						
113	0	0	10	0.027±0.008	0.3131	0.6
123	26	0.232±0.031	211	0.589±0.026	43.458	0.000
125	84	0.750±0.042	112	0.312±0.024	67.058	0.000
127	2	0.017±0.002	25	0.069±0.013	4.2629	0.045
D13S175						
101	3	0.026±0.012	21	0.058±0.01	1.793	0.300
103	8	0.071±0.021	88	0.245±0.02	15.875	0.000
105	91	0.812±0.036	157	0.438±0.02	47.866	0.000
107	1	0.008±0.007	30	0.083±0.01	7.763	0.005
109	6	0.053±0.024	38	0.106±0.01	2.783	0.100
111	0	0	7	0.019±0.007	2.222	0.150
113	3	0.026±0.01	17	0.047±0.01	0.897	0.064
D13S143						
126	1	0.008±0.007	0	0	3.192	0.04
128	1	0.008±0.007	5	0.013±0.006	0.169	0.65
130	90	0.80±0.048	283	0.79±0.021	0.088	0.81
132	6	0.05±0.026	26	0.07±0.013	0.490	0.54
134	12	0.11±0.016	37	0.10±0.013	0.012	0.89
136	2	0.017±0.021	3	0.008±0.004	0.727	0.55
138	0	0	4	0.011±0.005	3.278	0.05

*D13S141. *The marker D13S141 has seven allelic variants [9, 12]; however, only four of them were revealed in the ethnic groups from the Volga-Ural region. The allele 123 (D13S141) frequency is significantly higher (χ ^2 ^ = 43.458; *р* = 0.000) on chromosomes of individuals from the control group (59%), whereas in NSHL patients, it is only 23%. The allele 125 (D13S141) is observed in the mutant chromosomes with a frequency of 75%, which is significantly higher than in normal chromosomes (31%) (χ ^2 ^ = 67.058; *р* = 0.000), and it matches the data on the allele 125 (D13S141) dominanceon chromosomes with the mutation с.35delG in NSHL patients from Morocco, Greece, Palestine, and Israel [9, 10, 12, 45].

**Table 4:** Frequencies of haplotypes D13S141-D13S175-D13S143, identified in chromosomes of patients with the с.35delG / с.35delG genotype and in normal chromosomes of healthy donors living in the Volga-Ural region

Haplotypes	Patients with c.35delG\c.35delG		Overall control		Russians		Tatars		Bashkirs	
	Absolute value	Frequency	Absolute value	Frequency	Absolute value	Frequency	Absolute value	Frequency	Absolute value	Frequency
113-101-130	1	0.009	1	0.002	0	0	1	0.008	0	0
123-101-130	1	0.009	12	0.033	4	0.023	7	0.056	1	0.016
125-101-130	0	0	4	0.011	4	0.023	0	0	0	0
113-101-134	1	0.009	0	0	0	0	0	0	0	0
125-101-136	0	0	1	0.002	1	0.006	0	0	0	0
123-103-128	0	0	1	0.002	0	0	1	0.008	0	0
113-103-130	0	0	3	0.008	2	0.012	1	0.008	0	0
123-103-130	2	0.018	28	0.078	13	0.076	12	0.097	3	0.048
125-103-130	1	0.009	20	0.055	12	0.070	4	0.032	4	0.064
127-103-130	0	0	5	0.013	4	0.023	0	0	1	0.016
113-103-132	0	0	1	0.002	0	0	1	0.008	0	0
123-103-132	0	0	1	0.002	0	0	1	0.008	0	0
125-103-132	0	0	2	0.005	1	0.006	1	0.008	0	0
123-103-134	3	0.027	8	0.022	2	0.012	4	0.032	2	0.032
125-103-134	0	0	3	0.008	3	0.017	0	0	0	0
125-103-136	1	0.009	0	0	0	0	0	0	0	0
123-103-138	0	0	1	0.002	0	0	1	0.008	0	0
125-105-128	1	0.009	0	0	0	0	0	0	0	0
123-105-128	2	0.018	0	0	0	0	0	0	0	0
113-105-130	1	0.009	3	0.008	3	0.017	0	0	0	0
123-105-130	8	0.071	64	0.178	25	0.145	24	0.194	15	0.242
125-105-130	66	0.590	35	0.097	24	0.140	5	0.040	6	0.097
127-105-130	0	0	3	0.008	3	0.017	0	0	0	0
113-105-132	1	0.009	0	0	0	0	0	0	0	0
123-105-132	1	0.009	3	0.008	0	0	2	0.016	1	0.016
125-105-132	4	0.036	1	0.002	1	0.006	0	0	0	0
127-105-132	0	0	2	0.005	2	0.012	0		0	0
123-105-134	3	0.027	9	0.025	3	0.017	4	0.032	2	0.032
125-105-134	5	0.045	4	0.011	3	0.017	1	0.008	0	0
127-105-134	0	0	1	0.002	0	0	1	0.008	0	0
123-105-136	0	0	2	0.005	0	0	2	0.016	0	0
125-105-138	0	0	1	0.002	0	0	1	0.008	0	0
123-105-138	0	0	1	0.002	1	0.006	0	0	0	0
113-107-130	0	0	1	0.002	0	0	1	0.008	0	0
123-107-130	1	0.009	16	0.044	4	0.023	4	0.032	8	0.129
125-107-130	0	0	1	0.002	1	0.006	0	0	0	0
123-107-128	0	0	1	0.002	0	0	0	0	1	0.016
125-107-128	0	0	1	0.002	0	0	1	0.008	0	0
127-107-130	0	0	2	0.005	0	0	2	0.016	0	0
123-107-132	0	0	1	0.002	0	0	1	0.008	0	0
123-107-134	0	0	1	0.002	0	0	1	0.008	0	0
125-109-126	1	0.009	0	0	0	0	0	0	0	0
123-109-130	1	0.009	16	0.044	9	0.052	4	0.032	3	0.048
125-109-130	2	0.018	7	0.019	6	0.035	1	0.008	0	0
127-109-130	0	0	1	0.002	1	0.006	0	0	0	0
123-109-132	0	0	2	0.005	1	0.006	1	0.008	0	0
125-109-132	0	0	1	0.002	1	0.006	0	0	0	0
127-109-134	1	0.009	1	0.002	0	0	0	0	1	0.016
125-109-134	0	0	1	0.002	1	0.006	0	0	0	0
123-109-134	0	0	1	0.002	1	0.006	0	0	0	0
123-109-136	1	0.009	0	0	0	0	0	0	0	0
125-109-138	0	0	1	0.002	1	0.006	0	0	0	0
123-111-130	0	0	3	0.008	0	0	3	0.024	0	0
127-111-130	0	0	2	0.005	1	0.006	0	0	1	0.016
123-111-134	0	0	1	0.002	0	0	1	0.008	0	0
125-111-134	0	0	1	0.002	0	0	0	0	1	0.016
123-113-130	2	0.018	8	0.022	6	0.035	1	0.008	1	0.016
125-113-130	1	0.009	3	0.008	2	0.012	1	0.008	0	0
123-113-134	0	0	1	0.002	0	0	0	0	1	0.016
Haplotypic diversity	0.645		0.943		0.944		0.917		0.946	
Total, chromosomes	112		358		172		124		62	
Number of haplotype variants	25		52		32		32		16	

*D13S175. *The marker D13S175 has eight allelic variants [9, 10, 12], of which seven are present in the ethnic groups of the Volga-Ural region. The allele 105 (D13S175) on c.35delG chromosomes is observed at a frequency of 81.2%, which is significantly higher in comparison with the normal chromosomes (43.8%) (χ ^2 ^ = 47.866; *р* = 0.000), and allele 103 (D13S175) was significantly more frequent in the controls (χ ^2 ^ = 47.866; *р* = 0.000 and χ ^2 ^ = 15.87; *р* = 0.000, respectively) ( *Table 3* ). Earlier, it was shown that, on the chromosomes with the mutation с.35delG in NSHL individuals from Tunisia, Algeria, Morocco, and Greece, allele 105 (D13S175) was also very frequent (from 67 to 100%) [9, 12, 46]. Allele 111 (D13S175) was not observed on the chromosomes of individuals with NSHL but it was present in 2% of the chromosomes of people with no hearing problems.

**Fig. 4:**
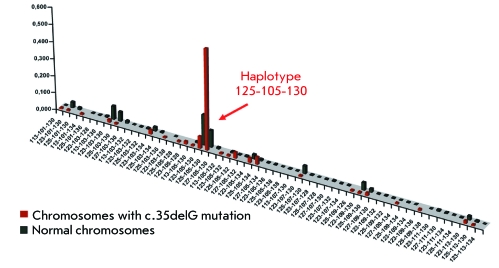
The distribution of frequencies of haplotype D13D141-D13S175-D13S143 on normal chromosomes and chromosomes with a mutation c.35delG in patients with NSHL (non-syndromic sensorineural hearing loss). Along the vertical and horizontal axes the frequency of the haplotype and the names of the haplotype are indicated, respectively. The arrow shows the haplotype 125-105-130.

*D13S143* **.** The marker D13S143 has eight allelic variants [9, 12], of which seven are present in the ethnic groups residing in the Volga-Ural region. Allele 130 (D13S143) is the most frequent both on normal chromosomes (79%) and c.35delG chromosomes (80%), and allele 134 (D13S143) is more often detected on chromosomes of с.35delG-mutant individuals (χ ^2 ^ = 9.909; *р* = 0.005). 

An analysis of the distribution of the allele frequencies of three microsatellite loci D13S141, D13S175, and D13S143 on normal and с.35delG chromosomes revealed a pronounced misbalance in linkage between the specific alleles of these markers and mutation с.35delG in the *GJB2* gene ( *Table 3* ). The degree of association of the out-gene microsatellite loci under study vividly reflects the standard coefficient of the allele association (∆ *St* ) [39]. The greatest degree of linkage with the mutation с.35delG is typical of allele 125 of the marker D13S141 (∆ *St* = -0.438) and allele 105 of the marker D13S175 (∆ *St* = -0.386). 

**Haplotype analysis and age of с.35delG mutation**

Given the data we obtained during the study of the polymorphism of the markers D13S141, D13S175, and D13S143, and the linkage disequilibrium of some of their alleles with the mutation с.35delG in the *GJB2* coding region, we suggested that they may be evidence of the presence of a single ancestor haplotype, which carries this mutation. As a result, for three polymorphic loci, haplotypes of members of each of the 56 families with hereditary deafness and healthy donors were constructed. A precise identification of the haplotype by the alleles D13S175–D13S143–D13S141 is possible for 112 mutant chromosomes with с.35delG and 358 normal chromosomes.

In all of the chromosomes analyzed, 59 different variants of haplotypes were revealed, of which 52 were found on normal chromosomes and 25 on с.35delG-mutant chromosomes ( *Table 4* ). 

The distribution of the haplotypes on 358 normal chromosomes is characterized by a high value of haplotypic diversity ( *h* = 0.943), the frequency of the most wide-spread haplotype 123-105-130 amounts to 17.8%, and 11 other haplotypes are found at frequencies exceeding 2%. For the distribution of haplotype frequencies on 112 chromosomes with the mutation c.35delG, a lower value of the haplotypic diversity ( *h* = 0.645) is typical, the haplotype 125-105-130 is the most frequently found (59%), and the frequency of six haplotypes exceeds 2%. Seven haplotypes rarely occurring on the mutant chromosomes (below 2%) were not detected on normal chromosomes. The graphic mapping of the occurrences of the haplotypes D13S141–D13S175–D13S143 on the normal chromosomes of healthy donors and с.35delG-mutant chromosomes in NSHL patients is illustrated in *Fig. 4* .

An analysis of the distribution of the haplotypes D13S141–D13S175–D13S143 on normal chromosomes in people of different ethnic origins (Russians, Tatars, and Bashkirs) showed differences in the spectrum of haplotypes in the surveyed ethnic groups and statistically significant differences in the frequencies of the haplotypes (χ ^2 ^ = 57.335; *р* = 0.000; d.f. = 56). In Russians (172 analyzed chromosomes), 32 of the 59 haplotypes were revealed in the total sample; in Tatars (124 chromosomes analyzed), 32 of the 59; and in Bashkirs (62 chromosomes), 17 of the 59 haplotypes.

**Table 5:** Association and linkage disequilibrium of markers D13S141, D13S175, and D13S143 with the mutation с.35delG

Marker	Allele	Р (95% significance level)	χ^2^	Δ
D13S141	125	<0.001	67.05872	0.636179
D13S175	105	<0.001	47.8665	0.666045
D13S143	130	0.81	0.08801	0.062381

**Table 6:** The number of generations which passed since the time of the c.35delG mutation spread in the Volga-Ural region

Marker	Number of generation since the time of the mutation’s appearance in the population (q)	Number of years which passed from the start of expansion	Start of expansion
D13S175 (allele 105)	470	11800	9800 B.C.
D13S141 (allele 125)	133	3300	1300 B.C.
Mean value	301	7500	5500 B.C.

In Russians, the most widespread haplotypes were 123-105-130 (14.5%), 125-105-130 (14.0%), and 123-103-130 (7.6%), while the remaining 29 occurred at different frequencies: from 0.6% (12 haplotypes) to 7.0% (haplotype 125-103-130). On normal chromosomes in Tatars, the haplotypes 123-105-130 and 123-103-130 occurred most often at frequencies of 19.4 and 9.7%, respectively, and the frequencies of the remaining 30 varied from 0.8% (19 haplotypes) to 5.6% (haplotype 123-101-130). In Bashkirs, the haplotypes 123-105-130 (24.2%), 123-107-130 (12.9%) and 125-105-130 (9.7%) were most often recorded, and their total frequency reached 46.8%. The frequencies of the remaining 14 haplotypes varied from 1.6% (9 haplotypes) to 6.4% (haplotype 125-103-130). Besides, in each ethnic group, specific haplotypes (D13S141–D13S175–D13S143), non-occurring in other groups, were observed at low frequencies: in Russians, 14 haplotypes; in Tatars, 15; and in Bashkirs, 4.

An analysis of the haplotypes D13S141-D13S175-D13S143 revealed that there is a higher frequency of haplotype 125-105-130 on chromosomes with the с.35delG mutation compared to the chromosomes of healthy donors (χ ^2 ^ = 64.866, *р * < 0.001), as well as an ethnic specificity of the spectra and frequencies of occurrence of the haplotypes D13S141–D13S175–D13S143 in three ethnic groups of healthy donors.

*Table 5*shows data on the association and linkage disequilibrium of marker D13S175, D13S143, and D13S141 alleles carrying the mutation с.35delG.

The values of the linkage disequilibrium parameter were greatest in alleles 105 and 125 of D13S175 and D13S141 markers, respectively, located proximally relative to the gene *GJB2* , and the lowest in alleles of the distal D13S143 marker. Stemming from the values of χ ^2 ^ and the linkage disequilibrium parameter δ, the most probable founder haplotype (the ancestor haplotype) seems to consist of the alleles 125-105-130 ( *Fig. 4* ). The haplotype 125-105-130 was revealed on 59% of all chromosomes carrying с.35delG, which is reliably higher ( *р * < 0.001) than the frequency of this haplotype (9.7%) on normal chromosomes.

To calculate the number of generations since the start of the spread of the с.35delG mutation in the populations of the Volgo-Ural region, we selected two markers: D13S175 and D13S141. The selection criterion for these markers was relatively high values of χ ^2 ^ and measure of linkage disequilibrium δ; statistically significant differences in the frequency distribution of these marker alleles in chromosomes with and without с.35delG were also considered.

No statistically significant differences in the frequency distribution of alleles of the marker D13S143 between c.35delG and intact chromosomes ( *Table 3* ), and concurrently a minimum value of the linkage disequilibrium parameter, were observed ( *Table 5* ). The prevalence of the allele 130 of this STR-marker in two groups of chromosomes (with and without the с.35delG mutation) formally explains the absence of statistically significant differences. Nevertheless, given the statistically significant differences obtained for two other STR-markers, which are closer to the с.35delG mutation, the existence of the ancestor haplotype for с.35delG, embracing the area covered by the D13S141-D13S175-D13S143 markers, and its subsequent “tailing” at the expense of recombination and mutation events during the numerous generations seem likely. However, the absence of a statistically significant association of the most frequent allele 130 (D13S143) and the ancestor haplotype provided grounds for excluding D13S143 from markers using which the number of generations was computed ( *Table 6* ).

After the beginning of divergence of the с.35delG-mutant ancestor haplotype in the populations of the Volga-Ural region, from 133 to 470 generations (on average 301 generations) passed. At the estimate of the age (in years) of the ancestor haplotype reconstructed on the territory of the Volga-Ural region, the duration of one generation, as in other studies, was considered equal to 25 years ( *Table 6* ).

The time over which the expansion of the с.35delG-mutant chromosomes has been present within the population of the Volga-Ural region ranges from 3,300-11,800 years (mean is ~ 7,500). However, such estimates of the number of generations (based on the physical distance) often lead to the overestimation of the “age” of the mutation, because it is the probable (not the observed) value for the mutation events that is considered; therefore, with this approach, researchers do not orientate mean values. Instead, they choose the most distant marker, which is linked however to the locus of the disease, i.e. the so-called boundary of the stable haplotype [47]. In the given case, it is the D13S175 marker. If one presumes that the number of generations calculated with the use of this marker is more accurate, then the most likely time of expansion of the founder haplotype with the mutation с.35delG in the populations of the Volga-Ural region is ~ 11,800 years. Such dating of the beginning of с.35delG expansion in the Volga-Ural region matches the results obtained in studies with the use of different DNA markers (SNP- and STR-markers) in other populations of Eurasia (10,000–14,000 years ago) [9–12].

The results of the haplotype analysis, estimates of the age of the c.35delG mutation, and data on the descending gradient to fit carrier frequency from south to north in the European populations allow to suggest that the Middle East and Mediterranean regions (probably, modern Greece) are the most probable centers of c.35delG origin, from where, together with Neolithic migrations of man, it widely propagated throughout Europe [9–12]. An analysis of the haplotypic diversity (using STR-markers) and approximate assessment of the age of c.35delG in the Volga-Ural region favor to a great extent the “traditional” Neolithic hypothesis about the origin and spread of this mutation. 

Considering the unified time continuum of the start of c.35delG spread in Eurasia, obtained with the use of various systems of DNA markers (SNP- and STR-markers), an assessment of the world haplotypic diversity of the mutant chromosomes accounting for the integral set of DNA markers is required to get an unambiguous answer to the question of the center of origin of the с.35delG mutation. 

## CONCLUSIONS

The analysis of the carrier frequency of the mutation c.35delG in the *GJB2* genein Eurasian populations has revealed a tendency towards gradient decrease in the c.35delG frequency from West to East, starting from the populations of Eastern Europe and the Volga-Ural region, with average frequencies of 3.3 and 1.4%, respectively; a low frequency (0.8–0.9%) in Central Asian populations, a minimum frequency (0.4%) in Yakuts residing in Eastern Siberia; and the absence of с.35delG in Altaians (Southern Siberia).

A haplotype analysis of the c.35delG-mutant chromosomes has allowed us to reconstruct the ancestor haplotype carrying this mutation and to confirm the unified origin of most of the studied mutant chromosomes of NSHL patients living in the Volga-Ural region. The estimated time of expansion of the c.35delG mutation carriers we obtained (11,800 years ago) fits the world values of the “age” of this mutation (10,000–14,000 years). 

The body of data collected should help clarify or re-view existing ideas about the center and time of origination of the c.35delG mutation ( *GJB2* ), as well as the factors that define its occurrence worldwide. 
